# Prevalence, Risk Factors, Characteristics, and Clinical Outcomes of Thrombocytopenia in the Intensive Care Unit: A Prospective Single‐Center Cohort Study

**DOI:** 10.1155/ccrp/4492230

**Published:** 2026-03-09

**Authors:** Zainab A. Almardod, Kishore G. Sam, Eman E. Younis

**Affiliations:** ^1^ Department of Pharmacy Practice, Dubai Medical University, Dubai, UAE; ^2^ Pharmaceutical Care Department, Saudi German Hospital—Madinah, Madinah, Saudi Arabia

**Keywords:** bleeding, drug-induced thrombocytopenia, heparin-induced thrombocytopenia, intensive care unit, platelets, thrombocytopenia

## Abstract

**Background:**

Thrombocytopenia is a common hematologic abnormality in the intensive care unit (ICU), affecting approximately 50% of patients. It is associated with increased mortality and bleeding risk. Despite its clinical significance, epidemiological studies on ICU‐related thrombocytopenia in Gulf Cooperation Council countries remain limited.

**Methods:**

This prospective observational cohort study was conducted to investigate the prevalence, risk factors, characteristics, and clinical outcomes of thrombocytopenia in the ICU. We included ICU patients admitted for ≥ 24 h, excluding pregnant women and individuals under 18. Thrombocytopenia was defined as a platelet count < 150 × 10^9^/L after ruling out pseudothrombocytopenia. Patients were stratified by thrombocytopenia severity and followed until discharge, death, or 30 days post onset. Risk factors were analyzed using multivariable modified Poisson regression models. The Naranjo probability scale and the 4Ts score were used for causal assessment of drug‐induced thrombocytopenia (DIT) and heparin‐induced thrombocytopenia (HIT). Primary outcomes included three‐month ICU mortality and major bleeding. Kaplan–Meier with log‐rank tests assessed time‐to‐mortality.

**Results:**

The study enrolled 276 patients; 38.8% had thrombocytopenia, including 23.4% with severe thrombocytopenia. The incidence of new‐onset thrombocytopenia was 22.5%. DIT was suspected in 15% of cases, including five patients with potential HIT. Shock diagnosis was a significant predictor of new‐onset thrombocytopenia (adjusted risk ratio = 2.26, 95% confidence interval: 1.35–3.79). Thrombocytopenic patients had higher Acute Physiology and Chronic Health Evaluation (APACHE) IV scores (*p* < 0.001) and experienced more major bleeding events (16.8% vs. 8.3%, *p* = 0.03) and higher mortality rates (27.1% vs. 5.3%, *p* < 0.001) with reduced time‐to‐mortality (log‐rank *p* = 0.01). New‐onset thrombocytopenia was independently associated with major bleeding and mortality.

**Conclusion:**

Thrombocytopenia is prevalent in the ICU, correlating to disease severity, major bleeding, and mortality. The study’s findings underscore the need for timely recognition and effective management of thrombocytopenia to improve patient outcomes.

## 1. Introduction

Thrombocytopenia is a frequently encountered hematological abnormality in intensive care unit (ICU) settings [1, 2]. The estimated prevalence ranges from 25% to 77% [3], with an incidence of 13%–44.1% [4] in critically ill patients. It is defined as a platelet count below 150 × 10^9^/L [5, 6]. ICU patients are particularly susceptible to new‐onset thrombocytopenia due to various factors such as comorbidities, critical illness, multiple organ dysfunction, and the need for hemodynamic support [4, 7, 8]. Consequently, these patients are at increased risk for bleeding, morbidity, and mortality [9]. Severe thrombocytopenia is especially associated with increased bleeding diathesis, particularly spontaneous and serious bleeding when platelet counts drop below 10 × 10^9^/L [2, 5, 10, 11]. Several studies also regard thrombocytopenia as an independent predictor of ICU mortality, irrespective of bleeding [4, 7, 12, 13]. Additionally, thrombocytopenia is associated with prolonged ICU and hospital length of stay (LOS) [3, 7, 13, 14]. It also correlates strongly with increased disease severity as indicated by higher scores on assessment tools such as the Acute Physiology and Chronic Health Evaluation (APACHE) scores, observed in thrombocytopenic critically ill patients [15–18].

Research has focused on identifying risk factors to predict and prevent complications related to thrombocytopenia. These factors include high disease severity, organ dysfunction (particularly renal and hepatic), sepsis, shock, disseminated intravascular coagulation, and trauma [3, 4, 8, 19, 20]. Other independent contributors involve large‐volume fluid resuscitation and massive blood transfusions, associated with dilutional thrombocytopenia [21]. Demographic and therapeutic contributors, such as older age, female sex, use of hemodialysis, inotropic agents, and certain medications (e.g., nonsteroidal anti‐inflammatory drugs, antibiotics, and chemotherapy) [3, 18], have also been implicated [4, 8, 19].

Identifying the etiology of thrombocytopenia is crucial for appropriate management, but often complex due to its multifactorial nature [7, 14]. Diagnosis involves a detailed review of patient history, medications, clinical presentation, and laboratory results [22, 23]. Pseudothrombocytopenia, a falsely low platelet count caused by EDTA‐induced clumping, must always be excluded [21, 24]. Common causes include infections, bleeding, hemodilution, liver dysfunction, and medications [2, 14]. Drug‐induced thrombocytopenia (DIT) can arise not only from antithrombotic and cytotoxic drugs but also from various antibiotics, such as linezolid, as well as older antiepileptics, such as valproic acid and quinine derivatives [25–27]. Prompt identification and management of the underlying cause are essential for improving outcomes.

Although thrombocytopenia in critically ill patients has been widely studied in Western and Asian populations, there is a lack of epidemiological data from the Gulf Cooperation Council (GCC) countries. To address this gap, we conducted a prospective study in the ICU of a tertiary hospital in Madinah, Saudi Arabia. We investigated the prevalence, risk factors, management strategies, and clinical outcomes of thrombocytopenia, along with the clinical characteristics of thrombocytopenic patients.

## 2. Methods

### 2.1. Study Design and Ethical Approval

This prospective observational cohort study was conducted in a 43‐bed adult ICU at a private tertiary hospital in Madinah, Saudi Arabia. Ethical approval was obtained from the Dubai Pharmacy College Institutional Review Board (IRB), Dubai, UAE (approval number: REC/PG/2023/13) on February 12, 2023. Subsequently, permission to conduct the study was granted by Saudi German Hospital—Madinah. The Ethics Committee waived the requirement for patient consent to collect, analyze, and publish anonymized data for this noninterventional study.

### 2.2. Participants

All adult patients admitted to the ICU for at least 24 h between March 16 and June 15, 2023, were eligible for inclusion. Pregnant women and individuals under 18 years were excluded. Patients with a platelet count below 150 × 10^9^/L at admission or at any point during their ICU stay constituted the thrombocytopenia cohort. Cases of pseudothrombocytopenia were excluded from the thrombocytopenia cohort and classified as nonthrombocytopenic.

### 2.3. Variables and Assessment

Baseline demographics and potential thrombocytopenia risk factors, including admission with sepsis or shock, renal or hepatic impairment, antimicrobial therapy, and antithrombotic use, were collected at ICU admission. APACHE IV and International Medical Prevention Registry on Venous Thromboembolism (IMPROVE)‐Bleeding Risk (BR) scores were calculated within the first 24 h of ICU admission. For thrombocytopenic patients, Sequential Organ Failure Assessment (SOFA) scores were also recorded periodically at 24, 48, and 96 h to assess organ dysfunction. Additional variables were collected to identify potential predictors of severe thrombocytopenia, including comorbidities, bleeding history, other hematologic abnormalities, and relevant past treatments. All medications administered at least one day before and continued during platelet decline were also reviewed to evaluate their association with thrombocytopenia incidence and severity.

The causal association of DIT and heparin‐induced thrombocytopenia (HIT) was evaluated using the Naranjo probability scale and the 4Ts score, respectively. To exclude alternative causes of thrombocytopenia, we thoroughly reviewed patients’ medical history, laboratory and imaging studies, and administered treatments. Management strategies for thrombocytopenic patients were reviewed, and the prognosis was determined based on the last recorded platelet count.

Patients were followed from the onset of thrombocytopenia until ICU discharge, death, or a maximum of 30 days. Thrombocytopenia was categorized according to the lowest platelet count as mild (100–149), moderate (50–99), severe (10–49), or very severe (< 10 × 10^9^/L). Due to the small number of very severe cases, these were combined with the severe category for analysis.

The primary outcomes were three‐month ICU mortality and major bleeding complications. We also recorded ICU LOS for up to 90 days.

### 2.4. Definitions

Thrombocytopenia was defined as a platelet count < 150 × 10^9^/L, after ruling out pseudothrombocytopenia [5, 6]. Pseudothrombocytopenia was identified based on either (1) a normal platelet count within 12 h of an abnormal result or (2) a ≥ 100 × 10^9^/L fluctuation in platelet counts across consecutive daily measurements. Patients developing thrombocytopenia during their ICU stay were designated as having new‐onset thrombocytopenia, while those with thrombocytopenia at admission were classified as having preexisting thrombocytopenia.

Renal impairment was defined as an estimated glomerular filtration rate < 80 mL/min/1.73 m^2^ or an established diagnosis of acute or chronic kidney disease (CKD). Hepatic impairment was defined as liver enzyme elevation more than three times the upper limit of normal or a documented diagnosis of liver cirrhosis, viral hepatitis, ischemic hepatitis, or traumatic liver injury. Major bleeding was defined as World Health Organization (WHO) grade 3 or 4 bleeding.

The causal association of DIT was assessed using the Naranjo probability scale. Scores of 5–8 (probable) or > 8 (definite) were considered diagnostic of DIT. In the absence of alternative etiologies, a score of 1–4 (possible) was also accepted to define DIT. HIT was determined by a 4Ts score of 4–5 (intermediate probability) or > 5 (high probability), together with a temporal relationship to heparin exposure or discontinuation. When no other causes of thrombocytopenia were plausible, a 4Ts score of < 4 (low probability) was also accepted.

### 2.5. Sample Size Estimation

Sample size was estimated a priori using Raosoft calculator [28] using a conservative expected thrombocytopenia prevalence of 25%, with a 95% confidence level and a 5% margin of error, yielding a target sample of 285 patients. As this was a prospective observational cohort study, all consecutively eligible ICU patients admitted during the study period were enrolled, resulting in a final sample of 276 patients, of whom 107 developed thrombocytopenia.

### 2.6. Statistical Analysis

All analyses were performed using IBM SPSS Statistics version 30.0 (IBM Corp., Armonk, NY, USA) and R software version 4.5.2 (R Core Team, 2025) within the RStudio environment (version 2025.09.2 + 418; Posit Team, 2025).

#### 2.6.1. Descriptive Statistics and Univariable Analysis

Continuous variables were assessed for normality using Shapiro–Wilk tests and visual inspection of distribution plots. As most variables were non‐normally distributed, they were summarized as medians with interquartile ranges (IQRs) and compared using the Kruskal–Wallis test. Categorical variables were expressed as frequencies and percentages and compared using Pearson’s Chi‐square test.

Univariable analyses were conducted to explore associations between clinical variables and overall thrombocytopenia, new‐onset thrombocytopenia, severe thrombocytopenia, mortality, and major bleeding outcomes. Risk ratios (RRs) with 95% confidence intervals (CIs) were calculated where appropriate using crosstabulation. Missing data were limited to a single observation, which was imputed using the median of neighboring observations.

The prevalence and incidence of thrombocytopenia were estimated with the corresponding 95% CIs using the one‐sample binomial test. Time‐to‐event outcomes were analyzed using the Kaplan–Meier method; differences between groups were compared using the log‐rank test. A two‐sided *p* *-*value < 0.05 was considered statistically significant.

#### 2.6.2. Multivariable Analysis

Multivariable analyses were performed using modified Poisson regression with generalized estimating equations (GEE) and robust sandwich variance estimators to estimate adjusted RRs (aRRs) and 95% CIs. Analyses were conducted using the *geeglm* function from the *geepack* package in R. An “independence” working correlation structure was specified for all models. This approach was selected over logistic regression because odds ratios tend to overestimate risks when outcomes are common (> 10%) in cohort studies [29].

Variables were selected for multivariable modeling based on clinical relevance and a univariable association threshold of *p* ≤ 0.20. For models of severe thrombocytopenia and mortality, a more conservative inclusion threshold of *p* ≤ 0.10 was applied to avoid model overfitting and improve estimate stability. In the mortality model evaluating the association with worsening thrombocytopenia, severe thrombocytopenia was excluded due to clinical and statistical overlap, which resulted in unstable estimates when both variables were included in the model.

#### 2.6.3. Model Validation and Diagnostics

Model fit was assessed using the normalized residual sum of squares, following the methodology recommended by Hagiwara and Matsuyama (2024) [30] and the quasi‐likelihood under the Independence Model Criterion. Multicollinearity was evaluated using the variance inflation factor, with values < 5 considered acceptable. Composite severity and bleeding risk scores, including APACHE IV, SOFA, and IMPROVE‐BR scores, were not included in multivariable analyses because they incorporate individual variables already entered into the models, including platelet count, which would introduce mathematical coupling and potential collinearity [31].

#### 2.6.4. Handling Sparse Data

We evaluated variables with sparse data distributions during model development. The variable “anemia” was excluded from the final multivariable models due to a zero frequency in one category, resulting in unstable parameter estimates and unreliable CIs, despite attempts to collapse categories and apply penalization.

## 3. Results

**FIGURE 1:**
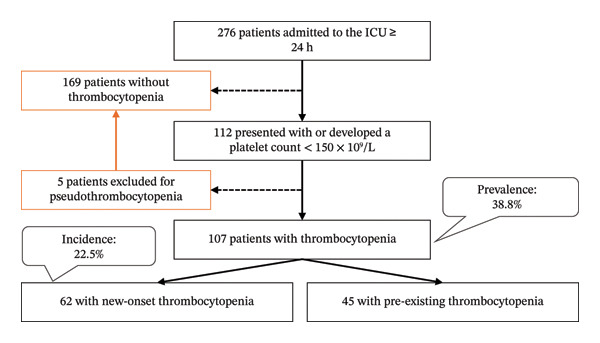
Study flowchart summarizing patient inclusion and the prevalence and incidence of thrombocytopenia. ICU, intensive care unit.

During the study period, 276 patients were admitted to the ICU for ≥ 24 h. Among them, 112 patients had at least one platelet reading < 150 × 10^9^/L. After excluding 5 patients with pseudothrombocytopenia, the final thrombocytopenia cohort included 107 patients, resulting in a prevalence of 38.8% (95% CI: 33%–44.8%) (Figure 1). The incidence of new‐onset thrombocytopenia was 22.5% (95% CI: 17.7%–27.9%). The median time from ICU admission to thrombocytopenia was 2 days (IQR: 1–5 days). The incidence of severe thrombocytopenia (platelet count < 50 × 10^9^/L) was 9.1% (95% CI: 5.9%–13.1%).

At initial measurement, 85% of thrombocytopenic patients had mild thrombocytopenia (Figure 2(a)). The median (IQR) initial low platelet count was 133 (117–143) × 10^9^/L. At nadir, 56.1% remained in the mild range, while 20.6% had moderate thrombocytopenia, 19.6% had severe thrombocytopenia, and 3.7% had very severe thrombocytopenia. The median (IQR) nadir platelet count was 107 (52.6–126) × 10^9^/L (Figure 2(b)).

FIGURE 2Severity of thrombocytopenia at (a) the initial low platelet count and (b) the nadir platelet count.

**Figure:**
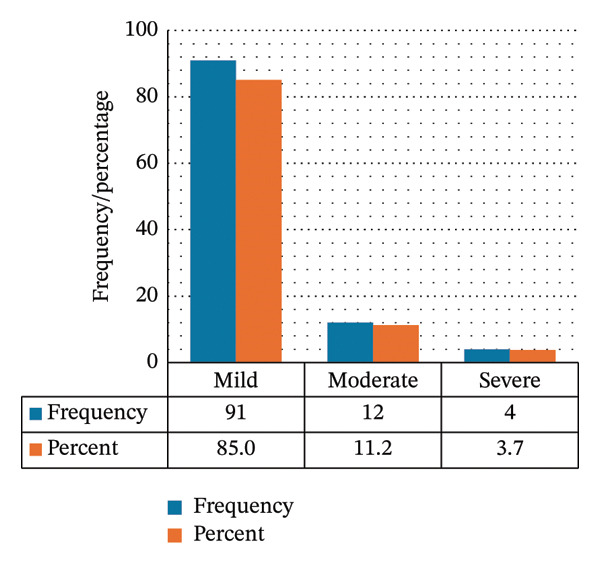
(a)

**Figure:**
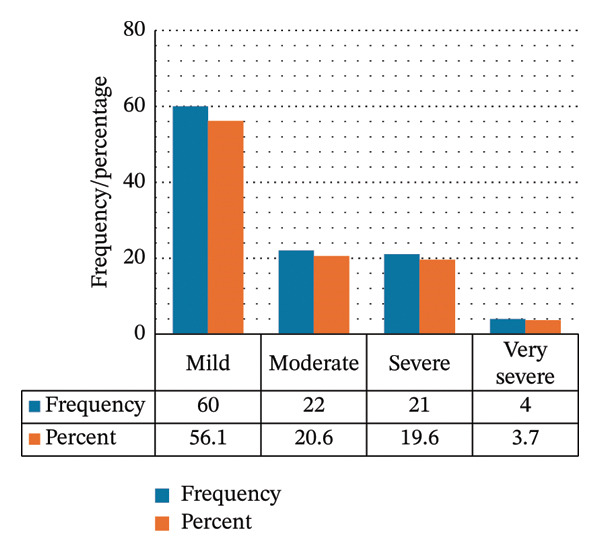
(b)

### 3.1. Baseline Characteristics and Thrombocytopenia Predictors

Baseline characteristics are summarized in Table 1. The median (IQR) age of thrombocytopenic patients was 64 (51–75) years; 65.4% were males. Most patients were admitted for medical conditions (81.3%), and 63.6% were Saudi nationals. There were no statistically significant differences in demographics between thrombocytopenic and nonthrombocytopenic patients.

**TABLE 1:** Baseline characteristics of patients with and without thrombocytopenia.

Characteristics	Thrombocytopenia				No thrombocytopenia *N* = 169
	Total *N* = 107	*p*‐value^a^	New‐onset *N* = 62	*p*‐value^a^	
Demographics					
Male, N (%)	70 (65.4)	0.18	39 (62.9)^c^	0.45	97 (57.4)
Age (years), median (IQR)	64 (51–75)	0.146	63.5 (44.8–76)^c^	0.36	62 (45–73)
Type of admission					
Medical, N (%)	87 (81.3)	0.81	47 (75.8)^c^	0.83	132 (78.1)
Surgical, N (%)	4 (3.7)		2 (3.2)		7 (4.1)
Trauma, N (%)	16 (15)		13 (21)		30 (17.8)
Nationality/geographical region					
Saudi, N (%)	68 (63.6)	0.2	38 (61.3)^c^	0.16	120 (71)
Not Saudi, N (%)	39 (36.4)		24 (38.7)		49 (29)
Asian, N (%)	22 (20.6)		14 (22.6)		
Middle Eastern, N (%)	9 (8.4)		5 (8.1)		
African, N (%)	7 (6.5)		4 (6.5)		
European, N (%)	1 (0.9)		1 (1.6)		
Clinical significant diagnosis on admission					
Shock (septic and others), N (%)	18 (16.8)	< 0.001	9 (14.5)^c^	0.003	6 (3.6)
Sepsis (without shock), N (%)	17 (15.9)	0.03	9 (14.5)^c^	0.12	13 (7.7)
Impaired renal function, N (%)	60 (56.1)	< 0.001	29 (46.8)^c^	0.1	59 (34.9)
Impaired liver function, N (%)	17 (15.9)	0.11	6 (9.7)^c^	0.96	16 (9.5)
Significant therapies on admission^b^					
Antimicrobial, N (%)			58 (93.5)^b^	0.06	142 (84)
Antithrombotic, N (%)			55 (88.7)^b^	0.06	131 (77.5)
Risk stratification scores					
APACHE IV score, median (IQR)	47 (33–62)	< 0.001	47 (30–66.3)^c^	0.009	36 (27–49.5)
IMPROVE‐BR score, median (IQR)	8 (6–10.6)	< 0.001	7.3 (5–9.5)^d^	0.003	6 (5–8.5)
Outcomes					
Major bleeding, N (%)	18 (16.8)	0.03	13 (21)^c^	0.008	14 (8.3)
3‐month ICU mortality, N (%)	29 (27.1)	< 0.001	22 (35.5)^d^	< 0.001	9 (5.3)
ICU LOS (days), median (IQR)	5.2 (2.8–13)	< 0.001	8.4 (3–17.5)^d^	< 0.001	3 (2–6)

*Note:* Categorical variables were tested by Pearson’s Chi‐square test, while continuous variables were tested by the Kruskal–Wallis test.

Abbreviations: APACHE, Acute Physiology and Chronic Health Evaluation; ICU, intensive care unit; IMPROVE‐BR, International Medical Prevention Registry on Venous Thromboembolism‐Bleeding Risk; IQR, interquartile range; LOS, length of stay.

^a^Compared to patients without thrombocytopenia.

^b^Evaluated only for new‐onset thrombocytopenia, as those with preexisting thrombocytopenia have not received those therapies yet.

^c^ *p* > 0.05 when compared to patients with preexisting thrombocytopenia.

^d^ *p* < 0.05 when compared to patients with preexisting thrombocytopenia.

Patients with thrombocytopenia were frequently admitted with shock (16.8, *p* < 0.001), sepsis (15.9%, *p* = 0.03), and renal impairment (56.1%, *p* < 0.001). They had significantly higher APACHE IV and IMPROVE‐BR scores than nonthrombocytopenic patients (both *p* < 0.001) (Table 1). There was no statistically significant difference in APACHE IV scores between patients with new‐onset and preexisting thrombocytopenia (*p* > 0.1). However, IMPROVE‐BR scores were higher in patients with preexisting thrombocytopenia than those with new‐onset thrombocytopenia (9 [7–11.5] vs. 7.3 [5–9.5], *p* = 0.01).

In multivariable analysis, shock (aRR = 2.14, 95% CI: 1.56–2.92), sepsis (aRR = 1.49, 95% CI: 1.03–2.16), and renal dysfunction (aRR = 1.49, 95% CI: 1.10–2.03) remained statistically significant for overall thrombocytopenia (Table 2). For new‐onset thrombocytopenia, only shock remained statistically significant (aRR = 2.26, 95% CI: 1.35–3.79, *p* = 0.002).

**TABLE 2:** Multivariable analysis of thrombocytopenia predictors by the modified Poisson regression model.

Risk factors	aRR (95% CI)	Multivariable *p*‐value
(A) All thrombocytopenia cases (*N* = 107)		
Age	1.00 (0.996–1.01)	0.37
Male sex	0.75 (0.56–1.01)	0.06
Saudi nationals	0.77 (0.57–1.03)	0.08
Shock diagnosis (septic and other types)	2.14 (1.56–2.92)	**< 0.001**
Sepsis diagnosis (without shock)	1.49 (1.03–2.16)	**0.04**
Impaired renal function	1.49 (1.10–2.03)	**0.01**
Impaired liver function	1.12 (0.79–1.59)	0.5
(B) New‐onset thrombocytopenia (*N* = 62)		
Saudi nationals	0.71 (0.47–1.06)	0.09
Shock diagnosis (septic and other types)	2.26 (1.35–3.79)	**0.002**
Sepsis diagnosis (without shock)	1.58 (0.89–2.80)	0.12
Impaired renal function	1.23 (0.80–1.91)	0.35
Antimicrobial treatment	1.75 (0.66–4.65)	0.26
Antithrombotic treatment	1.66 (0.80–3.42)	0.17

*Note:* The bold values indicate statistical significance (*p* < 0.05).

Abbreviations: aRR, adjusted risk ratio; CI, confidence interval.

### 3.2. Identification of DIT

DIT was suspected in 16 patients (15%). Suspected causative drugs are listed in Table 3. Seven DIT cases involved potential drug interaction: clopidogrel with aspirin (*N* = 5), linezolid with sulfasalazine (*N* = 1), and heparin with aspirin (*N* = 1). Based on 4Ts scores, HIT was suspected in five patients (4.7%): three received enoxaparin and two received unfractionated heparin.

**TABLE 3:** Drugs suspected of causing DIT.

Drug classes	Drug names	N (% of DIT)
Antiplatelet, *N* = 6	Clopidogrel (with aspirin)	5 (31.3)
	Ticagrelor	1 (6.3)

Anticoagulant (HIT), *N* = 5	Enoxaparin	3 (18.8)
	Unfractionated heparin	2 (12.5)

Antimicrobial, *N* = 2	Linezolid	1 (6.3)
	Piperacillin	1 (6.3)

Thrombolytic, *N* = 1	Alteplase	1 (6.3)

Antineoplastic (chemotherapy/targeted therapy), *N* = 2	Chemotherapy	1 (6.3)
	Dasatinib	1 (6.3)

Abbreviations: DIT, drug‐induced thrombocytopenia; HIT, heparin‐induced thrombocytopenia.

### 3.3. Clinical Characteristics and Predictors of Severe Thrombocytopenia

Thrombocytopenic patients had multiple comorbidities (Table 4). Liver disease was observed in 24% of patients with severe thrombocytopenia (*p* = 0.01). All patients with severe thrombocytopenia had concurrent anemia (*p* = 0.01). Active bleeding at the time of platelet decline or a bleeding history (within 3 months) was present in 68% of severe cases (*p* = 0.045). Use of linezolid (*p* = 0.005) and levetiracetam (*p* = 0.02) was also significantly more frequent in severe thrombocytopenia cases. No statistically significant associations were found with other conditions or treatments.

**TABLE 4:** Clinical characteristics and predictors of severe thrombocytopenia.

Characteristics	Total cohort, *N* = 107 N (%)		Severe and very severe thrombocytopenia, *N* = 25				
			*N* (%)	Univariable		Multivariable	
				RR (95% CI)	*p*‐value	aRR (95% CI)	*p*‐value
Comorbid conditions							
Cardiovascular		71 (66.4)	16 (64)	0.90 (0.44–1.84)	0.78	—	—
Endocrine		67 (62.6)	16 (64)	1.06 (0.52–2.17)	0.87	—	—
Neurological		30 (28)	7 (28)	0.998 (0.47–2.14)	0.996	—	—
CKD		26 (24.3)	3 (12)	0.43 (0.14–1.31)	0.101	—	—
Respiratory		22 (20.6)	6 (24)	1.22 (0.55–2.69)	0.63	—	—
Liver disease		11 (10.3)	6 (24)	2.76 (1.41–5.40)	**0.01**	3.95 (1.71–9.10)	**0.001**
Hematologic/oncologic		6 (5.60	3 (12)	2.29 (0.95–5.54)	0.11	—	—
Other hematologic abnormalities							
Anemia^a^	90 (84.1)		25 (100)	Incalculable^a^	**0.01**	—	—
Leukopenia	13 (12.1)		4 (16)	1.38 (0.56–3.38)	0.5	—	—
Shock diagnosis	18 (16.8)		8 (32)	4.94 (2.39–10.24)	< 0.001	1.79 (0.88–3.64)	0.11
Sepsis diagnosis	17 (15.9)		4 (16)	1.56 (0.58–4.25)	0.6		
Active bleeding or within 3 months	54 (50.5)		17 (68)	2.09 (0.99–4.42)	**0.045**	1.33 (0.60–2.91)	0.48
Past treatment							
Cephalosporins	27 (25.2)		5 (20)	0.74 (0.31–1.78)	0.49	—	—
Piperacillin‐tazobactam	23 (21.5)		8 (32)	1.72 (0.85–3.47)	0.14	—	—
Meropenem	12 (11.2)		3 (12)	1.08 (0.38–3.07)	0.89	—	—
Levofloxacin	9 (8.4)		4 (16)	2.07 (0.91–4.72)	0.12	—	—
Linezolid	9 (8.4)		6 (24)	3.44 (1.86–6.35)	**0.001**	2.93 (1.37–6.30)	**0.006**
Vancomycin	7 (6.5)		1 (4)	0.60 (0.09–3.78)	0.56		
Heparin	18 (16.8)		7 (28)	1.92 (0.94–3.92)	0.09	1.56 (0.69–3.53)	0.28
Enoxaparin	34 (31.8)		8 (32)	1.01 (0.48–2.11)	0.98	—	—
Antiplatelet	26 (24.3)		5 (19.2)	0.78 (0.33–1.87)	0.57	—	—
Levetiracetam	11 (10.3)		6 (24)	2.76 (1.41–5.40)	**0.01**	2.87 (1.22–6.79)	**0.02**
Pantoprazole	49 (45.8)		15 (60)	1.78 (0.88–3.59)	0.103	—	—
Acetaminophen	23 (21.5)		7 (28)	1.42 (0.68–2.98)	0.37	—	—
Statin therapy	14 (13.1)		1 (4)	0.28 (0.04–1.89)	0.12	—	—
Furosemide	13 (12.1)		2 (8)	0.63 (0.17–2.36)	0.47	—	—
Fluid resuscitation	22 (20.6)		4 (16)	0.74 (0.28–1.92)	0.52	—	—
Blood products^b^	26 (24)		7 (28)	1.21 (0.58−2.54)	0.62	—	—
Surgery	14 (13.1)		1 (4)	0.28 (0.04–1.89)	0.12	—	—

Abbreviations: aRR, adjusted risk ratio; CI, confidence interval; CKD, chronic kidney disease; RR, risk ratio.

^a^Anemia was excluded from the final multivariable analysis due to quasi‐complete separation. This occurred because all cases of severe thrombocytopenia also presented with anemia, resulting in zero frequencies that prevented the stable estimation of confidence intervals. This is also the reason univariable RR with corresponding 95% CI could not be calculated.

^b^Blood products evaluated included packed red blood cells and fresh frozen plasma.

In multivariable analysis, independent factors associated with severe thrombocytopenia were liver disease (aRR = 3.95, 95% CI: 1.71–9.10), linezolid treatment (aRR = 2.93, 95% CI: 1.37–6.30), and levetiracetam treatment (aRR = 2.87, 95% CI: 1.22–6.79) (Table 4).

APACHE IV and IMPROVE‐BR scores increased with thrombocytopenia severity (Table 5, Figure 3). SOFA scores also rose over time, reaching a median (IQR) of 10 (6–13) at 96 h in patients with severe thrombocytopenia (*p* < 0.001). In contrast, SOFA scores decreased over time in patients with mild thrombocytopenia (Table 5, Figure 3(c)).

**TABLE 5:** Risk stratification scores and outcomes across severity groups.

	**Mild thrombocytopenia*, N* = 60**	**Moderate thrombocytopenia*, N* = 22**	**Severe and very severe thrombocytopenia, *N* = 25**	**p** **-value**

Risk stratification scores				
APACHE IV score, median (IQR)	40.5 (26–49)	57.5 (43.3–69.3)	62 (48.5–74.5)	< 0.001
IMPROVE‐BR score, median (IQR)	7 (5–9)	9 (7–11.1)	10.5 (8–13)	< 0.001
SOFA scores^a^, median (IQR)				
Initial (first 24 h)	5 (3–7)	8.5 (4.8–12)	7 (4–12)	< 0.001
At 48 h (*N* = 93)	4 (3–6)	7 (6–10.8)	9 (6–13)	< 0.001
At 96 h (*N* = 65)	4 (3–6)	9 (5–11)	10 (6–13)	< 0.001
Outcomes				
Major bleeding, N (%)	5 (8.3)	4 (18.2)	9 (36)	0.008
3‐month ICU mortality, N (%)	2 (3.3)	10 (45.5)	17 (68)	< 0.001
ICU LOS (days), median (IQR)	3.5 (2.4–7.1)	6.1 (3.4–14.9)	13 (7–34)	< 0.001

Abbreviations: APACHE, Acute Physiology and Chronic Health Evaluation; ICU, intensive care unit; IMPROVE‐BR, International Medical Prevention Registry on Venous Thromboembolism‐Bleeding Risk; IQR, interquartile range; LOS, length of stay; SOFA, Sequential Organ Failure Assessment.

^a^SOFA scores were not performed if the patient died or was discharged before the 3^rd^ day of admission, resulting in a smaller cohort with measured post‐48 and 96 h SOFA scores.

FIGURE 3Boxplots of the Kruskal‐Wallis test results for the (a) APACHE IV scores, (b) IMPROVE‐BR scores, and (c) SOFA scores at 96 h from the initial score, across the severity groups of thrombocytopenia. The boxes describe the interquartile ranges, with horizontal bars indicating the medians and vertical bars indicating the maximum and minimum distribution limits. The white dots indicate outliers. APACHE, Acute Physiology and Chronic Health Evaluation; IMPROVE‐BR, International Medical Prevention Registry on Venous Thromboembolism‐Bleeding Risk; SOFA, Sequential Organ Failure Assessment.

**Figure:**
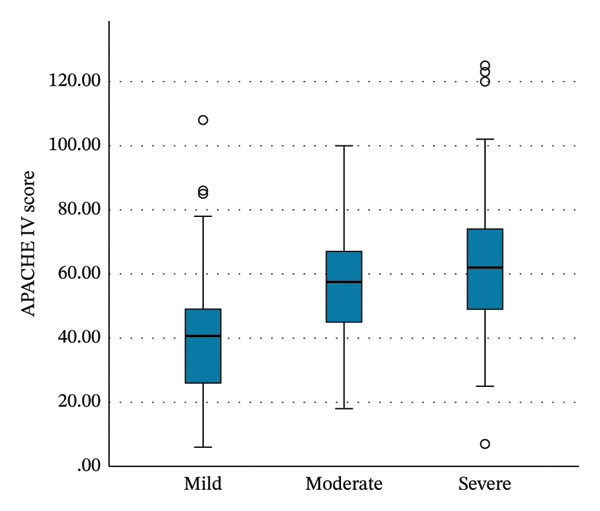
(a)

**Figure:**
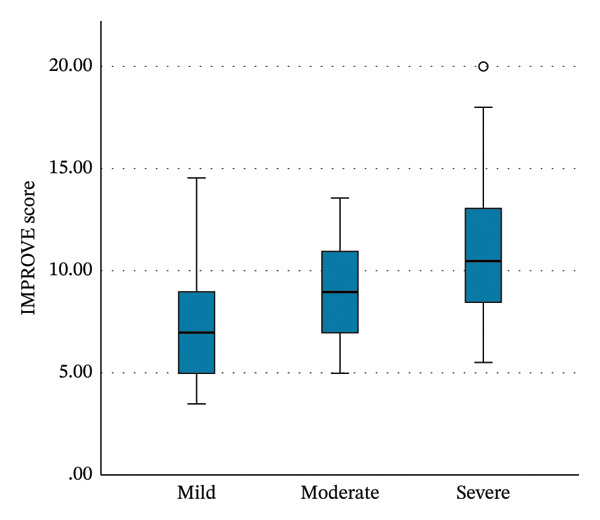
(b)

**Figure:**
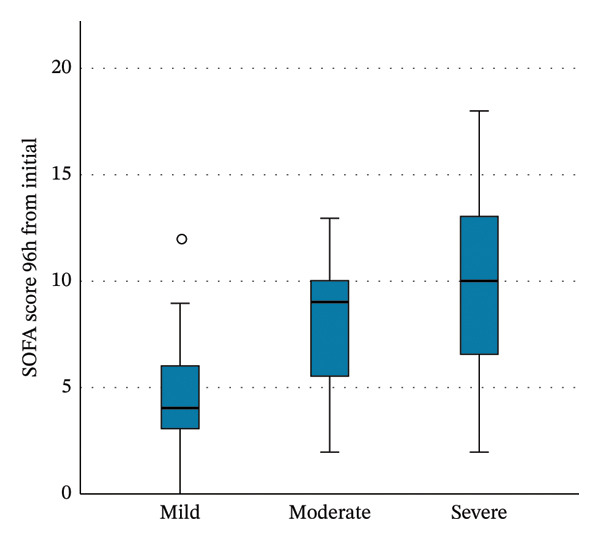
(c)

### 3.4. Management of Thrombocytopenia

Thrombocytopenia was managed conservatively in 56.1% of patients by addressing underlying causes. The most common active intervention was adjustment of drug therapy (36.4%), followed by platelet transfusions (12.1%) and tranexamic acid administration (4.7%). Multiple treatment strategies were used in 9.3% of patients.

Patients receiving no thrombocytopenia‐specific treatment had a *survival rate* of 88.3% (*p* = 0.001). In the treated groups, *mortality rates* were 53.8% (*p* = 0.006), 38.5% (*p* = 0.005), and 60% (*p* = 0.004) among patients who received platelet transfusions, those managed with medication adjustment, and those receiving multiple modalities, respectively. Treatment with tranexamic acid was not significantly associated with survival (*p* > 0.1).

### 3.5. Clinical Outcomes

#### 3.5.1. Bleeding Complications

Major bleeding occurred more frequently in thrombocytopenic patients than in nonthrombocytopenic patients (16.8% vs. 8.3%, *p* = 0.03), resulting in an increased risk of major bleeding (RR = 2.03, 95% CI: 1.05–3.91) (Table 1). In new‐onset thrombocytopenia, major bleeding occurred in 21% of patients (*p* = 0.008) with an RR of 2.53 (95% CI: 1.26–5.08). There was no significant difference in major bleeding between new‐onset and preexisting thrombocytopenia. Furthermore, major bleeding increased with thrombocytopenia severity (36% in severe cases, *p* = 0.008) (Table 5).

In multivariable analysis, overall thrombocytopenia was not an independent predictor of major bleeding (*p* > 0.1; Supporting Table S1). However, new‐onset thrombocytopenia remained significantly associated with major bleeding (aRR = 2.25, 95% CI: 1.08–4.66; *p* = 0.03).

#### 3.5.2. ICU LOS and Survival

Thrombocytopenic patients had longer ICU stays than nonthrombocytopenic patients (5.2 [2.8–13] vs. 3 [2–6] days, *p* < 0.001) (Table 1). The LOS was longer in patients with new‐onset thrombocytopenia (8.4 [3–17.5]) compared to patients without thrombocytopenia and patients with preexisting thrombocytopenia (3.8 [1.9–6.3]), *p* < 0.001 for both. ICU LOS increased with increasing thrombocytopenia severity (Table 5), reaching 13 7–34 days in severe cases (*p* < 0.001).

Kaplan–Meier analysis showed shorter time‐to‐mortality in the thrombocytopenia cohort (median 31 days, 95% CI: 0.62–61.38) and in the new‐onset thrombocytopenia group (median 19 days, 95% CI: 0.69–37.31). Median survival was not estimable in the nonthrombocytopenia cohort as mortality did not reach 50% (Figure 4). Log‐rank *p*‐values were 0.01 and 0.02, respectively (Table 6). Patients with moderate and severe thrombocytopenia had shorter time‐to‐mortality than those with mild thrombocytopenia, with a log‐rank *p* of 0.002 (Table 6).

FIGURE 4Kaplan–Meier curves of cumulative survival probability comparing (a) thrombocytopenia (blue/solid line) versus nonthrombocytopenia (green/dashed line) cases and (b) patients with mild (blue/solid line), moderate (green/dashed line), and severe (red/dotted line) thrombocytopenia. Tick marks (+) indicate censored observations (e.g., ICU discharge). Median survival times for nonthrombocytopenia and mild thrombocytopenia groups could not be determined within the study′s timeframe, as those curves did not drop below 0.5 probability. ICU, intensive care unit; LOS, length of stay.

**Figure:**
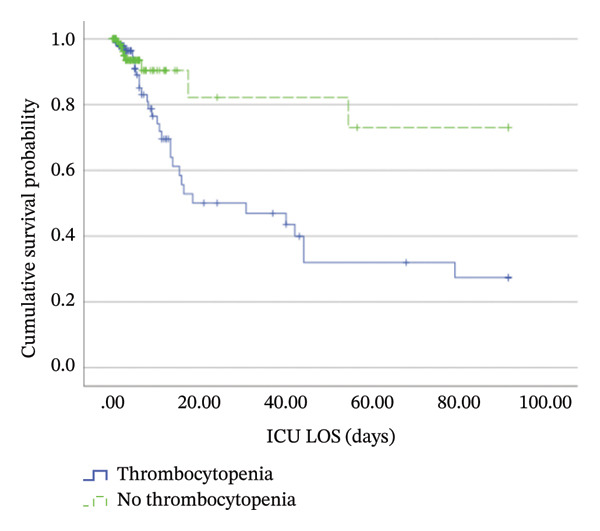
(a)

**Figure:**
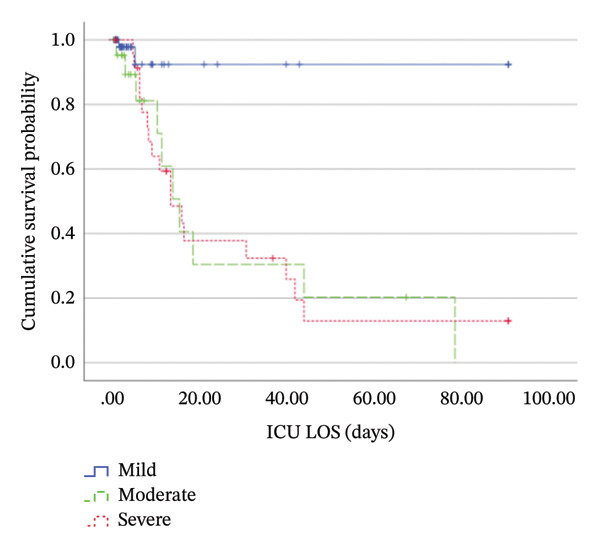
(b)

**TABLE 6:** Patients’ survival by Kaplan–Meier analysis.

Group	Time‐to‐mortality (days), median (95% CI)	Log‐rank *p*‐value
Thrombocytopenia		
Total (*N* = 107)	31 (0.62–61.38)	0.01**^∗^**
New onset (*N* = 62)	19 (0.69–37.31)	0.02**^∗^**
Severe (*N* = 25)	14 (7.34–20.66)	0.002**^∗∗^**
Moderate (*N* = 22)	16 (10.12–21.88)	
Mild (*N* = 60)	Not estimable^a^	
No thrombocytopenia (*N* = 169)	Not estimable^a^	

*Note:* The bold values indicate statistical significance (*p* < 0.05).

Abbreviation: CI, confidence interval.

^a^The median survival time cannot be estimated as mortality did not reach 50%.

^∗^Compared to patients without thrombocytopenia.

^∗∗^Compared to mild and moderate thrombocytopenia.

#### 3.5.3. Mortality and Thrombocytopenia Prognosis

The overall 3‐month ICU mortality during the study was 13.8%. Mortality was higher among thrombocytopenic patients (27.1% vs. 5.3%, *p* < 0.001; RR = 5.09, 95% CI: 2.51–10.33), particularly in those with new‐onset thrombocytopenia (35.5%, RR = 6.66, 95% CI: 3.25–13.67), compared to nonthrombocytopenic patients (Table 1). Mortality risk remained significant in multivariable analysis in the total thrombocytopenia cohort (aRR = 2.91, 95% CI: 1.36–6.24, *p* = 0.006) and in the new onset group (aRR = 3.88, 95% CI: 1.76–8.59, *p* < 0.001). Details on confounders are summarized in Table S1. Mortality increased with thrombocytopenia severity, 45.5% in moderate and 68% in severe cases, compared with 3.3% in mild thrombocytopenia (*p* < 0.001; Table 5). All patients with very severe thrombocytopenia (< 10 × 10^9^/L) died.

Thrombocytopenia resolved in 39.3% of patients (median recovery time: 4 [2–7.3] days). Deterioration occurred in 24.3%, while 36.4% had fluctuating or static counts or lacked follow‐up measurements due to discharge or death. Deterioration was more frequent in new‐onset thrombocytopenia than in preexisting thrombocytopenia (29% vs.17.8%, *p* = 0.006). Recovery time was longer in patients with severe thrombocytopenia (13 [6.8–27.3] days, *p* < 0.001) compared with mild (2 [1–7.9] days) and moderate thrombocytopenia (7.5 [4.4–14] days). Thrombocytopenia deterioration was significantly associated with a mortality rate of 76.9% (RR = 9.79, 95% CI: 4.73–20.25, *p* < 0.001). This remained significant after adjusting for age, sex, new‐onset thrombocytopenia, major bleeding, liver impairment, shock, and ICU LOS (aRR = 6.69, 95% CI: 2.99–14.96, *p* < 0.001).

## 4. Discussion

In this prospective cohort study of predominantly medical critically ill patients, the prevalence of thrombocytopenia was 38.8% (95% CI: 33.0%–44.8%), and the incidence of new‐onset thrombocytopenia was 22.4% (95% CI: 17.7%–27.9%). Severe thrombocytopenia occurred in 9.1% (95% CI: 5.9%–13.1%) of ICU patients. Thrombocytopenia was associated with increased major bleeding events, longer ICU LOS, and higher three‐month ICU mortality, particularly among patients with new‐onset and severe thrombocytopenia. After multivariable adjustment, new‐onset thrombocytopenia remained independently associated with major bleeding and mortality.

Our prevalence and incidence estimates align with existing literature reporting thrombocytopenia rates in ICU populations between 37.6% and 46% and new‐onset thrombocytopenia between 16.5% and 30% [8, 9, 18, 20, 32]. Similarly, the rate of severe thrombocytopenia was comparable to some studies [17, 20], while other investigators reported lower rates [8, 18]. These variations likely reflect differences in case mix and ICU admission criteria.

Thrombocytopenia in the ICU is multifactorial, commonly attributed to the increased severity of illness, organ dysfunction, and severe infection, leading to increased consumption, impaired production, and immune‐mediated platelet destruction [2, 6, 7, 12, 33, 34]. Various studies correlated lower platelet counts with higher APACHE scores, indicating higher disease severity [16, 17, 35, 36]. In our study, thrombocytopenic patients had higher APACHE IV scores, which increased with thrombocytopenia severity. Similarly, SOFA scores increased over time, especially with severe thrombocytopenia. This reinforces the link between declining platelet counts and progressive organ dysfunction.

We also assessed bleeding risk at ICU admission using the IMPROVE‐BR score. Thrombocytopenic patients had higher scores corresponding to severity, especially those with preexisting thrombocytopenia. This is likely because platelet count < 50 × 10^9^/L is incorporated into the IMPROVE‐BR score. Although primarily validated for thromboprophylaxis‐related bleeding risk, our findings suggest that the IMPROVE‐BR score may have utility in bleeding risk stratification for thrombocytopenic ICU patients [37, 38].

The clinical impact of bleeding in critically ill patients extends beyond hemostasis alone, as it may lead to hemorrhagic shock, massive transfusion, and interruption of essential therapies like antithrombotics [39, 40]. Although the relationship between thrombocytopenia and bleeding remains inconsistent, our findings support other studies correlating thrombocytopenia with a higher risk of *major bleeding* [13, 17, 20]. In contrast, some studies found no significant association [9, 32].

Consistent with previous studies, thrombocytopenic patients had higher mortality rates and longer ICU stays with shorter time‐to‐mortality, particularly those with severe or new‐onset thrombocytopenia [3, 7, 13, 14, 20]. Conversely, Anthons et al. [20] observed a higher mortality rate among patients with preexisting thrombocytopenia. One study found no association between thrombocytopenia and ICU or hospital LOS [9]. Importantly, worsening thrombocytopenia emerged as an independent predictor of ICU mortality, supporting prior observations [16, 35]. This may explain the increase in mortality in patients with new‐onset thrombocytopenia, as more patients in this subgroup experienced worsening thrombocytopenia.

Among identified risk factors, admission with shock was the only independent predictor of new‐onset thrombocytopenia. Meanwhile, sepsis, shock, and renal impairment were associated with overall thrombocytopenia. Past studies commonly reported septic shock, increased severity of illness, and organ dysfunction, particularly renal and hepatic, as key risk factors for thrombocytopenia [3, 4, 8, 19]. Unlike some prior studies, we found no significant associations with age or sex [3, 13, 20].

Liver disease, linezolid, and levetiracetam treatment were independently associated with severe thrombocytopenia. These associations should be interpreted as hypothesis‐generating rather than causal. Thrombocytopenia is common in chronic liver disease, primarily attributed to reduced thrombopoietin production and splenic sequestration [41]. Linezolid is associated with dose‐ and time‐dependent reversible myelosuppression, particularly in patients with renal impairment [42–45]. Some studies reported higher rates of severe thrombocytopenia with linezolid compared to vancomycin [46], whereas others found no worsening of baseline thrombocytopenia in critically ill patients treated with linezolid [47]. Levetiracetam is associated with rare and reversible thrombocytopenia, possibly by immune‐mediated mechanisms [48, 49]. In our study, alternative etiologies were identified, suggesting confounding factors may have contributed to observed associations. For instance, severe infections in patients treated with linezolid could impair platelet function [50]. While with levetiracetam, severe systemic illness or underlying neurological injury, such as stroke and traumatic brain injury, may have played a role [51]. Further research is required to clarify these relationships.

DIT was suspected in 15% of patients based on our defined criteria (see Methods), including five cases with possible HIT. Diagnosing DIT in the ICU is challenging due to overlapping etiologies and limited confirmatory testing [2, 52]. Our observed proportion of suspected DIT is comparable to prior reports [9]. Additionally, in our cohort, potential contributing drugs included clopidogrel, ticagrelor, alteplase, piperacillin, linezolid, dasatinib, and chemotherapy, consistent with existing literature [53–59]. Several reports have linked clopidogrel and ticagrelor to severe thrombocytopenia, particularly thrombotic thrombocytopenic purpura [60–66], while the drug interaction between linezolid and sulfasalazine was suspected due to the common myelosuppressive property of both agents [24, 47, 54, 67, 68].

Management of thrombocytopenia was primarily supportive, leading to better survival. Adjusting medications suspected of causing thrombocytopenia or increasing bleeding risk was the most common intervention, which correlated with improved survival. Platelet transfusions did not improve survival, likely reflecting underlying disease severity in transfused patients [69]. Tranexamic acid was infrequently used and was not associated with a survival benefit, though it remains a potential adjunct in select bleeding patients [70–72].

In this study, we did not distinguish between prophylactic and therapeutic platelet transfusions or assess their impact on bleeding. However, recent multicenter studies suggest that most ICU platelet transfusions are prophylactic [20, 73], although some report a lower proportion [69]. Notably, prophylactic transfusions have not shown improved thrombocytopenia or survival [73, 74]. Current guidelines recommend restrictive prophylactic transfusion strategies [10, 70, 75, 76]. The European Society of Intensive Care Medicine recommends prophylactic platelet transfusion at platelet counts < 10 × 10^9^/L [75], while the Australian Blood Management Guidelines suggest a threshold of < 20 × 10^9^/L [76].

Our findings have several practical implications. First, patients with thrombocytopenia should be evaluated for reversible causes, including medication review. Closer platelet monitoring is recommended for patients with sepsis, shock, or renal impairment, as worsening thrombocytopenia in septic patients is linked to higher mortality [35, 77]. Second, clinicians should consider platelet counts before prescribing medications associated with thrombocytopenia, especially in high‐risk patients. Lastly, decisions on prophylactic platelet transfusion should not rely solely on platelet thresholds but also consider bleeding risk, clinical context, and potential transfusion‐related complications [73, 74].

This study has several strengths, including its prospective design and adequate sample size, and focus on a population underrepresented in thrombocytopenia research: critically ill patients in the Gulf region. Moreover, the ICU in Madinah serves a diverse population, which may enhance the external validity of our findings.

Our study also has some limitations. First, the single‐center design may limit generalizability due to variability in case mix and prescribing practices across different ICUs. Second, as an observational study, we lacked systematic confirmatory tests for DIT, such as antibody assays, drug levels, or rechallenge, which may have led to misclassification. Finally, although we used the IMPROVE‐BR score to estimate bleeding risk, this tool has not been specifically validated in ICU thrombocytopenia populations.

## 5. Conclusion

Thrombocytopenia is a common and clinically significant finding in the ICU, associated with greater illness severity, increased bleeding risk, prolonged ICU stay, and higher mortality rates, particularly in patients with new‐onset or severe thrombocytopenia. Key risk factors included sepsis, shock, and renal impairment, while certain medications like linezolid and levetiracetam were associated with greater thrombocytopenia severity. Routine platelet monitoring, early identification of reversible causes, and careful medication selection may help mitigate adverse patient outcomes.

Larger multicenter studies and randomized controlled trials are essential to establish evidence‐based management strategies tailored to ICU populations. We particularly encourage collaborative research across GCC countries to better define the epidemiology, causes, and optimal management of thrombocytopenia in critically ill patients in the region.

## Funding

This research received no external funding.

## Conflicts of Interest

The authors declare no conflicts of interest.

## Supporting Information

Supporting Table S1 presents the univariable and multivariable analyses of risk factors for mortality and major bleeding outcomes using a modified Poisson regression model. Separate models were constructed for overall thrombocytopenia and new‐onset thrombocytopenia, including relevant clinical covariates. The table reports risk ratios (RR and aRR) with corresponding 95% CIs and *p*‐values for each model.

## Supporting information


**Supporting Information** Additional supporting information can be found online in the Supporting Information section.

## Data Availability

The data that support the findings of this study are available on reasonable request from the corresponding author. The data are not publicly available due to privacy concerns and ethical restrictions.
