# Supplementary material for: Prevalence, Risk Factors, Characteristics, and Clinical Outcomes of Thrombocytopenia in the Intensive Care Unit: A Prospective Single‐Center Cohort Study
Source: Crit Care Res Pract. 2026 Mar 9;2026:4492230. doi: 10.1155/ccrp/4492230 (PMC12968733; doi:10.1155/ccrp/4492230)
Supplement: Supplementary file 1 — Supporting Information Additional supporting information can be found online in the Supporting Information section. [file CCRP-2026-4492230-s001.docx]

| **Table S1. Mortality and major bleeding risk factors by the modified Poisson regression model.** | | | | | | | |  |
| --- | --- | --- | --- | --- | --- | --- | --- | --- |
| **Characteristic** | **Univariable** | | **Multivariable** | | | | |  |
|  | **RR (95% CI)** | ***P*-value** | **Model with overall thrombocytopenia** | | | **Model with new-onset thrombocytopenia** | | |
|  |  |  | **aRR (95% CI)** | ***P*-value** | **aRR (95% CI)** | | ***P*-value** |  |
| 1. **Major bleeding risk factors** | | | | | | | |  |
| Age | 1 (0.98–1.02) | 0.92 | - | - | - | | - |  |
| Male sex | 1.96 (0.91–4.20) | 0.07 | 1.64 (0.82–3.28) | 0.16 | 1.67 (0.84–3.30) | | 0.14 |  |
| Overall thrombocytopenia | 2.03 (1.06–3.91) | **0.03** | 1.2 (0.58–2.49) | 0.62 | - | | - |  |
| New-onset thrombocytopenia | 2.53 (1.26–5.08) **^a^**  1.89 (0.72–4.92) **^b^** | **0.008 ^a^**  0.18 **^b^** | - | - | 2.25 (1.08–4.66) **^a^**  6.13 (2.42–15.49) **^b^** | | **0.03 ^a^**  **< 0.001 ^b^** |  |
| Shock diagnosis | 3.50 (1.77–6.92) | **< 0.001** | 2.63 (1.17–5.94) | **0.02** | 3.90 (1.81–8.38) | | **< 0.001** |  |
| Sepsis diagnosis | 0.27 (0.04–1.87) | 0.13 | 0.30 (0.04–2.23) | 0.24 | 0.41 (0.06–2.95) | | 0.37 |  |
| Impaired renal function | 0.79 (0.40–1.56) | 0.5 | - | - | - | | - |  |
| Impaired liver function | 2.06 (0.97–4.39) | 0.07 | 1.03 (0.42–2.52) | 0.94 | 1.20 (0.51–2.82) | | 0.68 |  |
| Antithrombotic treatment | 0.22 (0.12–0.44) | **< 0.001** | 0.25 (0.12–0.52) | **< 0.001** | 0.15 (0.08–0.28) | | **< 0.001** |  |
| ICU LOS | 1.01 (1.004–1.02) | **0.001** | 1.01 (0.999–1.02) | 0.07 | 1.01 (0.997–1.02) | | 0.14 |  |
| 1. **Mortality risk factors** | | | | | | | |  |
| Age | 1.04 (1.02–1.06) | **< 0.001** | 1.03 (1.01–1.04) | **0.003** | 1.02 (1.01– 1.04) | | **0.008** |  |
| Male sex | 1.00 (0.55–1.83) | 0.998 | - | - | - | | - |  |
| Thrombocytopenia | 5.09 (2.51–10.33) | **< 0.001** | 2.91 (1.36–6.24) | **0.006** | - | | - |  |
| New-onset thrombocytopenia | 6.66 (3.25–13.67) **^a^**  2.28 (1.07–4.87) **^b^** | **< 0.001 ^a^**  **0.02 ^b^** | - | - | 3.88 (1.76–8.59) **^a^**  2.34 (1.20–4.57) **^b^** | | **< 0.001 ^a^**  **0.01 ^b^** |  |
| Shock diagnosis | 6.13 (3.68–10.19) | **< 0.001** | 2.21 (1.04–4.70) | **0.04** | 2.27 (1.08–4.78) | | **0.03** |  |
| Sepsis diagnosis | 0.27 (0.04–1.87) | 0.29 | - | - | - | | - |  |
| Impaired renal function | 2.26 (1.22–4.18) | **0.007** | 1.33 (0.68–2.60) | 0.4 | 1.45 (0.76–2.75) | | 0.26 |  |
| Impaired liver function | 2.63 (1.41–4.91) | **0.003** | 1.45 (0.79–2.65) | 0.23 | 1.65 (0.81–3.36) | | 0.16 |  |
| Major bleeding | 4.45 (2.58–7.68) | **< 0.001** | 2.21 (1.12–4.36) | **0.02** | 1.81 (0.86–3.81) | | 0.12 |  |
| ICU LOS | 1.01 (1.001–1.02) | **< 0.001** | 1.01 (0.995–1.02) | 0.32 | 1.004 (0.994–1.02) | | 0.38 |  |
| Univariable analysis by Pearson’s Chi-square and multivariable analysis by modified Poisson regression.  **^a^** Compared to nonthrombocytopenic patients.  **^b^** Compared to pre-existing thrombocytopenia.  RR, risk ratio; aRR, adjusted risk ratio; CI, confidence interval; ICU, intensive care unit; LOS, length of stay. | | | | | | | |  |
